# Aptamers: A promising chemical antibody for cancer therapy

**DOI:** 10.18632/oncotarget.7178

**Published:** 2016-02-03

**Authors:** Gang Zhou, George Wilson, Lionel Hebbard, Wei Duan, Christopher Liddle, Jacob George, Liang Qiao

**Affiliations:** ^1^ Storr Liver Centre, Westmead Millennium Institute for Medical Research, University of Sydney and Westmead Hospital, Westmead, NSW, Australia; ^2^ Discipline of Molecular and Cell Biology, James Cook University, Townsville, QLD, Australia; ^3^ School of Medicine, Deakin University, Waurn Ponds, VIC, Australia

**Keywords:** aptamers, SELEX, siRNA, cancers, target therapy

## Abstract

Aptamers, also known as chemical antibodies, are single-stranded nucleic acid oligonucleotides which bind to their targets with high specificity and affinity. They are typically selected by repetitive *in vitro* process termed systematic evolution of ligands by exponential enrichment (SELEX). Owing to their excellent properties compared to conventional antibodies, notably their smaller physical size and lower immunogenicity and toxicity, aptamers have recently emerged as a new class of agents to deliver therapeutic drugs to cancer cells by targeting specific cancer-associated hallmarks. Aptamers can also be structurally modified to make them more flexible in order to conjugate other agents such as nano-materials and therapeutic RNA agents, thus extending their applications for cancer therapy. This review presents the current knowledge on the practical applications of aptamers in the treatment of a variety of cancers.

## INTRODUCTION

Aptamers are short, single-stranded oligonucleotides with unique three-dimensional configurations. Like conventional antibodies, aptamers bind to cognate targets and modulate their biological activities [1]. However, aptamers have several key advantages over conventional antibodies that make them promising therapeutic agents. For example, aptamers are smaller in size and therefore can more easily penetrate into the tumor core [2]. Aptamers are thermodynamically stable and lack immunogenicity, which enables their safe and effective retention in target cells *in vivo* [2]. Furthermore, aptamers can be synthesized *in vitro* independent of biological systems, thus eliminating the potential risk of bacterial or viral contamination, and importantly, they are flexible for structural and chemical modifications, eventually extending their clinical applications [3]. Given these features, aptamers have attracted a great deal of attention in cancer imaging, gene therapy and drug delivery. Some patented aptamers (such as A9 and A10) have been used as drug delivery vehicles for cancer therapy [4]. With the progress of aptamer selection technology, a number of novel aptamers that can regulate cell proliferation, signal transduction and immune function have been reported. In this article, we provide a comprehensive overview on recent progress and the therapeutic applications of aptamers in various cancers.

## APTAMER PRODUCTION

Aptamers are selected from a nucleic acid library followed by an *in vitro* screening process called SELEX [5]. Initially, a starting oligonucleotide pool containing a large number of random sequences (of the order of 10^14-15^) with a length of 22-100 nucleotides is designed. Two constant primer-binding sequences are found on both sides of the sequences so that they can be amplified by PCR. The SELEX process begins with the incubation of the library pool with target proteins. During incubation, only a very small portion of the library sequences can tightly bind to the target protein. Unbound or weakly bound sequences are then separated by various partitioning strategies. Sequences that specifically recognize targets are then eluted and amplified by PCR. The resulting PCR products form a new enriched library pool that can be used for subsequent rounds of SELEX. The process is repeated for several cycles to enrich the sequences that bind to targets with high affinity. Increased selection stringency is undertaken in the later rounds of SELEX by using effective competitors, decreasing the amount of proteins and increasing washing times. Typically, 8-18 rounds of SELEX are needed to obtain specific aptamer sequences [5, 6]. The resultant highly enriched sequences are then cloned, sequenced and chemically modified.

Significant progress has been achieved in aptamer-guided cancer therapy with the development of aptamers generated by cell-based SELEX, which uses living cells rather than the purified proteins as targets. Through cell-based SELEX, aptamers can be isolated without any prior knowledge of the molecular signatures of cell surface proteins [6]. At present, aptamers used in cancer therapy are classified into 3 parts: free aptamers against certain cancer specific proteins; free aptamers against immunoregulatory components; and aptamers as carriers for anti-tumor agents. Below we elaborate on the progress in each of these aspects.

## APPLICATION OF FREE APTAMERS IN TARGETED MOLECULAR CANCER THERAPY

**Figure 1:**
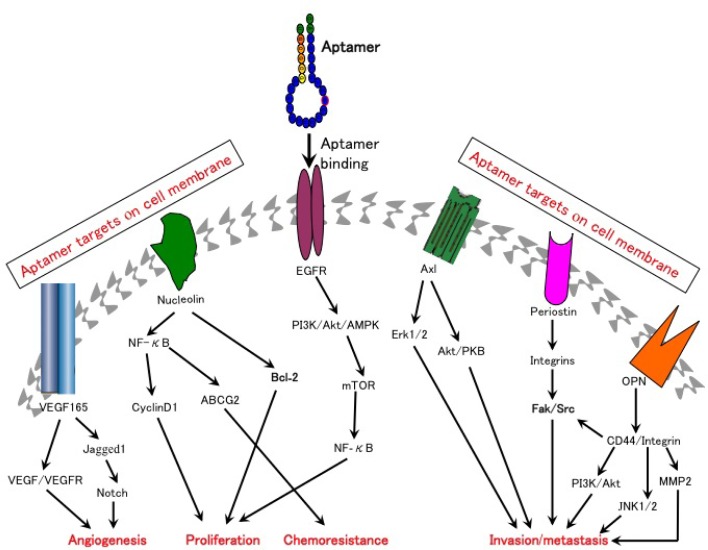
Aptamers can be used to target multiple molecular pathways that are critically involved in cancer development Aptamers ablate cell surface binding of their targets to the corresponding receptors and block targets-independent secondary downstream signaling pathways.

Abnormal activation of oncogenes or inactivation of tumor suppressor genes is believed to cause the dysregulation of key cellular pathways governing cell proliferation and apoptosis, resulting in the malignant transformation of stem cells and tumorigenesis [7]. Many monoclonal antibodies (mAbs) and small molecule inhibitors targeting tumor-driving proteins and aberrant molecular pathways are currently being tested for their anti-tumor effects in various cancers [7]. However, production of these agents is time- and labor-consuming, and costly, making their widespread use almost impossible. Owing to the advantages of aptamers, agonistic or antagonistic aptamers that are capable of activating or blocking key functional proteins possess great potential as novel substitutes for targeted cancer therapy (Table 1 and Figure 1).

**Table 1:** Aptamers (apt) explored for molecular-targeted cancer therapy

Apt (DNA/RNA)	Targets	Functions	Anti-tumor effect
Pegaptanib (RNA)	VEGF-165	Inhibits VEGF-associated tumor vessel formation	Potential therapeutic agent for solid cancers characterized by extensive angiogenesis [8]
ARC126/AX102 (RNA)	PDGF-B	Inhibits new blood vessel growth	Inhibits *in vivo* tumor angiogenesis of LLC and PIC [11,12]
SL (2)-B/RNV66 (DNA)	VEGF-165	Blocks VEGF angiogenesis	Inhibits *in vitro* cell proliferation of HCC [13] and Inhibit cell proliferation of BC *in vitro* and *in vivo* [14]
PPAR-apt (RNA)	PPAR	Inhibits PPAR-dependent VEGF signals	Inhibits *in vitro* tumor growth of CRC [15]
AS1411 (DNA)	Nucleolin	Inhibits nucleolin-associated cell processes and NF-κB or Bcl-2 signaling	Inhibits *in vitro* tumor growth of a variety of cancer cells; [17] Inhibits *in vivo* tumor growth of AML, LC, RC, BC and PAC;[16, 17] Shows superior anti-tumor activity in AML in clinical studies [18]
NOX-A12 (RNA)	CXCL12	Blocks CXCL12-induced cell migration and angiogenesis	Enhances HMCCs chemosensitization *in vivo*; [21] Reduces *in vivo* tumor burden of MM; Improves *in vivo* irradiation response of GBM; [22] Chemosensitizes CLL *in vitro* and *in vivo*; [23, 24] Improves CLL and MM clinical response
A30 (RNA)	HER3	Inhibits HRG-dependent tyrosine phosphorylation of HER2 and MAPK signaling	Inhibits *in vitro* tumor growth of BC cells and serves as a potential inhibitor for HER-3 over-expressed cancers such as LC, BC, GC and PAC [26]
E0727/CL428/KD11 30/TuTu2231 (RNA)	EGFR	Blocks EGFR phosphorylation and EGFR-mediated PI3K/AKT and MAPK signaling	Inhibits *in vitro* tumor proliferation of SQC [27], BC [30] and GBM; [29, 31] Induces *in vitro* cell apoptosis and inhibits *in vivo* LC growth [28]

Aptamers	Targets	Functions	Anti-tumor effect
E0727/A1532/U233/CL4 34(RNA)	EGFRVIII	Blocks autophosphorylation	Potential therapeutic agents for LC, BC [32, 33] Inhibit *in vitro* GBM cell proliferation and invasion [34]
Trimeric apt (DNA)	HER2	Stimulates ErbB-2 degradation	Inhibits *in vitro* and *in vivo* tumor growth of GC [35, 36]
PNDA-3 (DNA)	Periostin	Blocks interaction between periostin and its cell surface receptors	Inhibits tumor cell growth and metastasis *in vitro* and *in vivo* of breast cancer [39]
TTA140,41/GBI1042 (DNA)	TN-C	Blocks TN-C-involved pathways	Potential therapeutic agent for TN-C over-expressed cancers like GBM, BC, LC and CRC [40-42]
Apt-7 (RNA)	miRNA17/18a/19a/20a	Block the interaction of miRNA17-20a to target mRNAs	Inhibits cell proliferation of retinoblastoma tumor *in vitro* [44, 45]
NAS-24 (DNA)	Vimentin	Blocks cell proliferation	Causes *in vitro* and *in vivo* tumor apoptosis of adenocarcinomas [46]
YJ-1 (RNA)	CEA	Inhibits interaction of CEA and ribonucleoprotein M4	Inhibits *in vitro* migration and invasion of CRC and *in vivo* hepatic metastasis of CRC [47]
AFP-apt (RNA)	AFP	Inhibits AFP-involved pathways	Inhibits *in vitro* HCC proliferation [48]
Id1/3-PA7(Peptide)	Id1/Id3	Activates E-box promoter and increases CDKN2A expression	Induces *in vitro* cell-cycle arrest and apoptosis of OC [49] and BC [50]
AGE-apt (DNA)	AGE	Blocks interaction of AGE with RAGE	Inhibits *in vitro* and *in vivo* melanoma growth [51]
A-P50 (RNA)	NF-κB	Inhibits NF-κB activation	Prevents *in vitro* and *in vivo* tumor resistance to doxorubicin of LC [52]
GL21.T (RNA)	Axl	Blocks catalytic activity and Erk/Akt phosphorylation	Inhibits *in vitro* cell migration and invasion and *in vivo* tumor formation of LC [53]
OPN-R3 (RNA)	OPN	Blocks OPN-receptor binding	Inhibits *in vitro* cell migration and invasion and *in vivo* BC progression [54, 55]
KMF2-1a (RNA)	MCF-10AT	Targeting to BC cells	Potential therapeutic agent for BC [56]
AGC03/cy-apt (DNA)	HGC-27	Specifically binds GC cells	Potential therapeutic agent for GC [57, 58]
SDA (DNA)	E-/P-selectin	Blocks selectin-mediated cell adhesion	Inhibits *in vitro* tumor cell adhesion and metastasis of CRC [59]
TOV6 (DNA)	STIP1	Blocks binding of STIP1 to receptor	Inhibits *in vitro* cell invasion and metastasis of OC [60]
BC15 (DNA)	hnRNP A1	Blocks hnRNP A1 activation	Inhibits *in vitro* tumor cell proliferation and migration and *in vivo* tumor growth of HCCs [61]
P12FR2 (RNA)	PAUF	Blocks PAUF activation	Inhibits *in vitro* cell migration and *in vivo* tumor growth of PAC [62]
A9g (RNA)	PSMA	Blocks PSMA enzymatic activity	Inhibits PC *in vitro* and *in vivo* metastasis [63]
PSA-apt (RNA)	PSA	Blocks PSA-mediated signaling	Potential therapeutic agent for PC [64]
Heparanase-apt (DNA)	Heparanase	Inhibits enzymatic action of heparanase	Inhibits invasion of oral cancer cells *in vitro* [65]
rS3-PA (peptide)	STAT3	Reduces STAT3 phosphorylation and enhance its degradation	Inhibits tumor growth of GBM *in vitro* and *in vivo* [66]

Abbreviations: AFP: alpha fetoprotein; AGE: advanced glycation end; AML: acute myelocytic leukemia; Apt: aptamer; BC: breast cancer; Bcl-2: B-cell lymphoma 2; CDKN2A: cyclin-dependent kinase inhibitor 2A; CEA: carcinoembryonic antigen; CLL: chronic lymphocytic leukemia; COS: co-stimulatory; CRC: colorectal cancer; CTLA-4: cytotoxic T cell antigen-4; CXCL12: stroma cell-derived factor-1 ligand 12; GBM: glioblasoma; GC: gastric cancer; HCC: hepatocellular carcinoma; HMCCs: hematological cancer cells; hnRNP A1: heterogeneous nuclear ribonucleoprotein A1; HRG: heregulin; LC: lung cancer; LLC: Lewis lung carcinomas; MM: multiple myeloma; NF-κB: nuclear factor-κB; OC: ovarian cancer; OPN: osteopontin; PAC: pancreatic cancer; PAUF: pancreatic adenocarcinoma up-regulated factor; PC: prostate cancer; PIC: pancreatic islet cancer; PPAR: peroxisoe proliferator-activated receptor; PSA: prostate-Specific antigen; RAGE: AGE receptor; RC: renal cancer; SQC: squamous cell carcinoma; STIP1:stress-induced phosphoprotein 1; TN-C: tenascin-C.

### Aptamers targeting vascular endothelial growth factor (VEGF) and platelet-derived growth factor (PDGF)

The development of the first FDA-approved RNA aptamer pegaptanib (Macugen) to inhibit the binding of VEGF-165 to its receptor was a milestone in aptamer-based anti-angiogenesis therapies [8]. This aptamer was originally used for the treatment of age-related macular degeneration. Due to its excellent inhibitory effect on proliferation and permeability of endothelial cells, pegaptanib was subsequently considered a promising anti-angiogenesis agent for cancer therapy. However, anti-VEGF therapy alone has proved to be inadequate to inhibit new vessel formation in advanced stages of aberrant angiogenensis. This is likely due to the compensatory role of PDGF-B signaling in angiogenesis, as it has been reported that binding of PDGF-B to its receptor could recruit vascular mural cells (MCs, pericytes around capillaries and smooth muscles around large vessels) to endothelial cells (ECs), stimulate vessel maturation, and stabilize newly formed blood vessels [9]. It has also been reported that selective inhibition of the VEGF receptor (VEGF-R) resulted in the accumulation of MCs to tumor blood vessels where they could form an intimate vascular coverage, thus conferring resistance to the anti-VEGF-R reagents [10]. In order to overcome the resistance of anti-VEGF-R agents, an RTK inhibitor (SU6668) targeting both VEGFR-2 and PDGF-B was found to cause significant regression of C6 brain tumor vessels [9]. These studies imply that a combination of pegaptanib and anti-PDGF-B aptamers (ARC126/AX102) may have a synergistic effect on angiogenesis [11, 12]. Subsequently, another DNA aptamer against VEGF-165 was generated and modified with phosphorothioate linkages. This aptamer inhibited the proliferation of hepatocellular carcinoma cells and blocked Notch signaling by reducing the expression of its ligand Jagged1 [13] (Figure 1). A locked nucleic acid-modified DNA aptamer named RNV66 was recently found to directly target VEGF-165, resulting in efficient inhibition of breast cancer cell proliferation *in vitro* and *in vivo* [14]. In addition, a novel RNA aptamer targeting peroxisome proliferator-activated receptor δ (PPAR-δ), a transcription factor of VEGF-A, has been found to attenuate the tumor-forming ability of colorectal cancer cells [15]. Hence, aptamers targeting angiogenesis have great potential in cancer therapy.

### Aptamers targeting nucleolin and stromal cell-derived factor-1 ligand 12 (CXCL12)

Two aptamers AS1411 and NOX-A12 are currently in clinical trials for the treatment of patients with leukemia. Nucleolin is over-expressed in various cancers and is critical for cellular proliferation and apoptosis [16]. AS1411 is a guanine quadruplex aptamer that can bind to nucleolin, blocking the activation of secondary targets including nuclear factor-κB (NF-κB) and B-cell lymphoma 2 (BCL-2), therefore sensitizing the tumor cells to chemotherapy and improving their tumor-killing effects [16] (Figure 1). Pre-clinical studies, both *in vitro* and *in vivo*, have demonstrated that AS1411 exerts significant anti-tumor activity in many cancers [16, 17]. Clinical trials in patients with acute myelocytic leukemia (AML) demonstrated that the combination of AS-1411 with cytarabine results in superior therapeutic effects over cytarabine alone (Table 1) [18]. However, AS1411 only showed a marginal clinical response in a phase II trial in patients with renal cancer [19].

Another aptamer NOX-A12 which neutralizes CXCL12 (a critical chemokine involved in the homing and retention of hematological cancer cells) [20] was found to enhance the susceptibility of hematological cancer cells to conventional therapies, and inhibited the growth and metastasis of CXCL12-derived tumors [21-24]. As demonstrated in clinical trials, the combination of NOX-A12 and Bendamustine/Rituximab improved clinical responses in patients with chronic lymphocytic leukemia and multiple myeloma, as opposed to the single agents alone (clinical trials IDs: NCT01486797 and NCT01521533).

### Aptamers targeting the epidermal growth factor receptor (EGFR) family

The EGFR family contains four closely related receptor tyrosine kinases (RTKs) including HER1 (EGFR/ErbB-1), HER2 (ErbB-2), HER3 (ErbB-3) and HER4 (ErbB-4) [25]. Binding of epidermal growth factor (EGF) to EGFR leads to activation of EGFR kinase and subsequent autophosphorylation and activation of downstream signaling cascades [25]. Over-expression and/or mutations of the EGFR family are frequently found in several cancers including those of breast, brain, lung, colon and rectum, pancreas, and stomach. Thus, EGFR signaling can be an attractive target for cancer treatment [25]. In this perspective, A30, the first RNA aptamer against the EGFR family has been found to block the interaction of HER3 to its ligand (HRG), leading to the inhibition of breast cancer growth [26]. Subsequently, several anti-EGFR aptamers were generated and their tumor-suppressive functions have been demonstrated in a variety of cancers [27-31] (Table 1). Other aptamers that can specifically bind to EGFRVIII, a frequently identified mutant form of EGFR in breast cancer, lung cancer and glioblastoma, were shown to possess great potential as molecular anti-tumor reagents in cancers carrying the EGFRVIII mutation especially glioblastoma [32-34]. In addition, the trimeric aptamer against HER2 was found to be capable of engendering 2-fold stronger tumor inhibitory effects than that of HER2 antibodies in patients with gastric cancer [35, 36]. These data provide a solid foundation for developing aptamer-based approaches to target EGFR family in cancers.

### Aptamers targeting extracellular matrix (ECM) proteins

The extracellular matrix (ECM) is essential for cell adhesion and differentiation and functions as a major non-cellular component of tumor microenvironment or niche. It is demonstrated that disorganization or remodeling of ECM is proposed to promote cellular transformation and/or provide supportive tumorigenic microenvironment for cancer stem cells [37]. As such, therapeutic approaches that can target ECM components may hold promise in cancer treatment.

Periostin is an ECM component that is commonly over-expressed in a variety of cancers and is believed to be involved in cancer growth and metastasis [38]. A DNA aptamer termed as PNDA-3 was developed to block the interaction between periostin and its cell surface receptors (including integrins α_v_β_3_ and α_v_β_5_) [39]. Studies in breast cancer cells have demonstrated that PNDA-3 could significantly inactivate integrins α_v_β_3_- and α_v_β_5_-dependent signaling pathways and potently inhibit the growth and metastasis of breast cancer both *in vitro* and *in vivo* [39] (Figure 1). Moreover, PNDA-3 treatment can significantly attenuate the microvascular density of the xenograft tumors in mice. These results indicate that PNDA-3 may serve as a promising therapeutic candidate for cancers with abnormal periostin expression. Similarly, a modified RNA aptamer TTA1 against another ECM protein tenascin-C was tested in glioblastoma cells and later its efficient tumor uptake and durable blood retention were identified in mice bearing the xenograft tumors of breast, lung and colon, providing evidence that TTA1 may be potentially useful for tumor imaging and therapy [40-42].

### Aptamers targeting miRNAs

MicroRNAs (miRNAs) are short noncoding RNAs which can suppress gene expression and protein translation by binding to the complementary bases of target mRNAs and triggering mRNAs degradation [43]. The deregulated miRNAs such as miR-17-92 (miR-17, miR-18a, miR-19a, miR-20a, miR-19b and miR-92) oncogenic cluster can activate different tumorigenic signaling pathways or inhibit tumor-suppressive pathways, serving as critical biomarkers of many cancers such as those from breast, colon, lung, prostate and stomach (Figure 1) [43]. Therefore, targeting oncogenic miRNAs may shed a light for cancer treatment.

Current inhibitory strategies are mainly exerted by anti-miRNA oligonucleotides (AMOs), which target miRNAs through direct complementarity and preventing miRNA-mRNA combination. However, AMOs are inadequate to achieve satisfactory tumor inhibition since they can only target single miRNAs. By contrast, RNA aptamers that interact with their target RNAs rely on forming three-dimensional shape and are capable of binding to different sites within miRNAs cluster. This was successfully exemplified by a RNA aptamer (aptamer 7) that can bind to miRNA-18a, miRNA-19a and miRNA-20a through complementarity [44]. Aptamer 7 can accurately interfere with maturation and processing of target miRNAs. Intriguingly, aptamer 7 was also found to inhibit the processing of miRNA17 by additional weak interaction with its binding site located in close proximity to miRNA-19a. By inhibiting formation of mature miRNAs cluster (miRNA17-20a) and further up-regulating expression of corresponding target mRNAs, aptamer 7 was able to induce apoptosis and inhibit the proliferation of retinoblastoma cells *in vitro* [45]. The anti-tumor effect of the anti-miRNA aptamers is now being tested *in vivo*, and the current focus of anti-miRNA aptamers in cancer therapy includes the development of appropriate delivery tools and structural modifications for enhancing their therapeutic efficacy and stability in human blood.

### Aptamers targeting other proteins

Over recent years, therapeutically relevant aptamers against cell-surface proteins like vimentin, carcinoembryonic antigen (CEA), alpha fetoprotein (AFP) and aptamers against dyregulated signaling molecules such as NF-κB and STAT3 have been developed [46-66] (Table 1 and Figure 1). These aptamers show excellent binding affinity and specificity towards their targets, facilitating the development of novel molecular targeted cancer therapies.

## APTAMERS AGAINST IMMUNE REGULATORY PROTEINS

Effector T cells are an important part of anti-tumor immunity. T cells are activated by two types of signal presented by antigen presenting cells (APCs): (i) antigen-specific signals triggered by the T cell receptor (TCR), and (ii) signals induced by co-stimulatory (COS) molecules (such as 4-1BB, CD28 and OX40) [66]. Therefore, the interaction of COS molecules with their ligands is an important part of T cell activation. A reduction of COS ligands in APCs and the low antigenicity within tumors can compromise the ability of tumor-specific T cells to augment anti-tumor immunity [67]. Besides, APCs of tumor antigens convey immuno-suppressive signals to T cells through activating immuno-suppressive receptors and cytokines, driving T cells into a state of immune tolerance [67]. At present, some agonistic antibodies used as artificial COS ligands have achieved the desired COS effect in liver cancer, colorectal cancer and melanoma [68], however, their clinical application is hindered by manufacturing cost and potential immunogenicity. In contrast, aptamers have superior immunomodulatory effects over conventional antibodies in terms of synthesis cost and lack of immunogenicity (Table 2).

**Table 2:** Aptamers (apt) targeting the immune regulatory factors

Apt (DNA/RNA)	Targets	Functions	Anti-tumor effect
M12-23 (RNA)	4-1 BB	Interacts with 4-1 BB and induces COS signaling	Induces *in vitro* protective immunity of T cells and *in vivo* tumor regression of **mastocytoma [68]**
OX40-apt (RNA)	OX40	Induces nuclear translocation of NF-kB and IFN-γ production	Multimeric apt enhanced *in vivo* anti-tumor immunity of DC-based vaccines for melanoma [69,70]
CD28-apt (RNA)	CD28	Interacts with CD28 and provides artificial COS signaling	Costimulated CD4 and CD8 *in vitro* and elicited *in vivo* anti-tumor immunity against lymphoma [71]
Del60 (RNA apt)	CTLA-4	Blocks CTLA-4 function and stimulates T cell proliferation	Monomeric and multimeric apt inhibited *in vivo* tumor growth in melanoma and bladder tumor [72]
PSMA-4-1BB-apt (RNA)	PSMA/4-1BB	Delivers 4-1 BB COS ligand to PSMA (+) tumor cells	Inhibits *in vivo* tumor growth of CRC and *in vivo* lung metastasis of melanoma [73]
CD16*α/*c-Met-apt (RNA	CD16*α/*c-Met	Induces ADCC	Potential anti-tumor agents [74]
VEGF-4-1BB apt (DNA)	VEGF/4-1BB	Delivers 4-1 BB COS ligand to tumor stroma	Enhances *in vivo* vaccine-induced anti-tumor immunity and inhibits tumor growth of BC [75]
MP7 (DNA)	PD-1	Blocks PD-1/PD-L1 pathway	Inhibit *in vivo* tumor growth of colon cancer cells [76]
R5A1 (RNA)	IL10R	Blocks IL10 function	Inhibits *in vivo* tumor growth of CRC [77]
CL-42 (RNA)	IL4Rα	Blocks IL4Rα-STAT6 signaling	Inhibits *in vivo* tumor growth of mammary carcinoma [78]
IL-6 apt (DNA)	IL6	Blocks IL6-receptor interaction	Inhibit *in vitro* tumour cells proliferation of myeloma [79]
VR11 (DNA)	TNF-α	Inhibits binding of TNF-α to receptor	Potential non-immunogenic inhibitor of TNF-α [80]

Aptamer against 4-1BB (a COS receptor that is responsible for the activation and expansion of CD8^+^ T cells) was the first agonistic immunomodulatory aptamer. It acts as an artificial ligand for 4-1BB, and initiates COS signals to boost T cells survival. These aptamers have to form dimeric and/or multimeric aptamer structures in order to induce a 4-1BB oligomerization, a necessary process in the stimulation of receptor signaling [69]. Both dimeric and multimeric aptamers were equally efficient in stimulating T cells and inhibiting tumor growth as compared to the agonistic antibodies [69]. Using this approach, agonistic aptamers against OX40 and CD28, both of which are members of the tumor necrosis factor receptor (TNF-R) family, were subsequently developed and their multivalent derivatives demonstrated strong COS properties and greatly potentiated T cell dependent anti-tumor immunity in mice [70-72]. In addition, an antagonistic aptamer (Del 60) recognizing cytotoxic T cell antigen-4 (CTLA-4) (a membrane receptor that negatively regulates T cell activation) was used to reverse the CTLA-4-induced immune inhibitory signaling and facilitate antigen-dependent T cell proliferation. Similarly, its multivalent version resulted in enhanced anti-tumor immunity and tumor regression with comparable potency to that of CTLA-4 antibody [73].

Like other traditional anticancer drugs, immunostimulatory drugs also face the challenge of dose-limiting toxicity induced by non-specific targeting. In addition, immunostimulatory ligands may perturb the balance between self-antigen activated T cells (auto-reactive T cells) and regulatory T cells (Tregs), leading to increased auto-reactivity and consequently enhanced risks for adverse autoimmune pathology [74]. Thus, approaches that can prevent the access of immunostimulatory ligands to healthy cells and auto-reactive cells may be therapeutically relevant. In this regard, a bi-specific aptamer consisting of two aptamers targeting prostate specific membrane antigen (PSMA) and 4-1BB receptor was shown to activate T cells and inhibit the growth of PSMA-expressing tumors, while greatly minimizing drug toxicity and auto-immune reactions [74]. Similarly, a bi-specific aptamer targeting CD16*α* and c-Met was shown to recruit NK cells to c-Met-positive tumors and induce the production of CD16*α* which is an essential part of antibody-dependent cellular cytotoxicity (ADCC), thereby enhancing anti-tumor immunity [75]. Although data from clinical studies are still pending, the idea of using bi-specific aptamers to target cancers has shed light in developing novel strategies for cancer immunotherapy. However, for the bi-specific aptamer to work, the COS ligands have to be displayed on the cell surface to co-stimulate T cells. Thus, ligand-induced internalization of most receptors including PSMA into the cytosol can significantly restrict clinical applicability of this approach. In this aspect, targeting stroma-secreted products such as VEGF and osteopontin (OPN) rather than cell-surface expressed proteins such as PSMA and HER2 might be a more feasible approach [76].

Programmed death-1 (PD-1) is a Type I transmembrane protein of the Ig superfamily, and was found to be expressed on a number of activated immune cells such as T cells, B cells and NK cells [77], while its major ligand PD-L1 is frequently expressed on tumor cells. Interaction between PD-1 and PD-L1 was reported to inhibit the expression of many transcriptional factors of effector T cells and stimulate CD8^+^ cytotoxic T lymphocytes (CTL), leading to the apoptosis of tumor-infiltrating T cells [77]. In addition, the PD-1/PD-L1 axis also contributes to the differentiation of regulatory T cells (Tregs), which are critical inhibitors of the anti-tumor immune responses in the tumor microenvironment. Thus, PD-1/PD-L1 signaling is an important element in facilitating the immune evasion of tumors [77]. As such, the therapeutic efficacy of PD1/PD-L1 signaling inhibitors especially the mAbs against PD-1 (such as Nivolumab) has been tested in various stages of clinical trials in patients with non-small cell lung cancer, melanoma and renal cancer, but the outcomes were disappointing [77, 78]. Aptamer-based cancer targeting may be a promising and effective substitute of mAbs. For example, an aptamer against PD1/PD-L1 axis (MP7) was reported to inhibit PD-L1-induced apoptosis of tumor-specific T cells and IL-2 secretion. Furthermore, PEG-conjugated MP7 was shown to significantly inhibit the growth of colon cancer *in vivo* with the potency equivalent to that of anti-PD-L1 antibody. Although anti-PD-L1 antibodies have been approved for the treatment of melanoma and are being tested for other cancers (lung, ovarian and colon), the superiority of aptamer-based therapy for solid tumors should be further explored [79].

Aptamers acting as the antagonists of immune suppressive factors such as interleukin 10R (IL-10R), IL4Rα, IL-6 and TNF-α have also been developed. These aptamers are as effective as their respective antibodies in blocking the function of cognate targets and promoting protective immunity, representing a novel therapeutic alternative for cancer treatment [80-83].

## APTAMER-BASED DELIVERY OF THERAPEUTIC AGENTS

**Figure 2:**
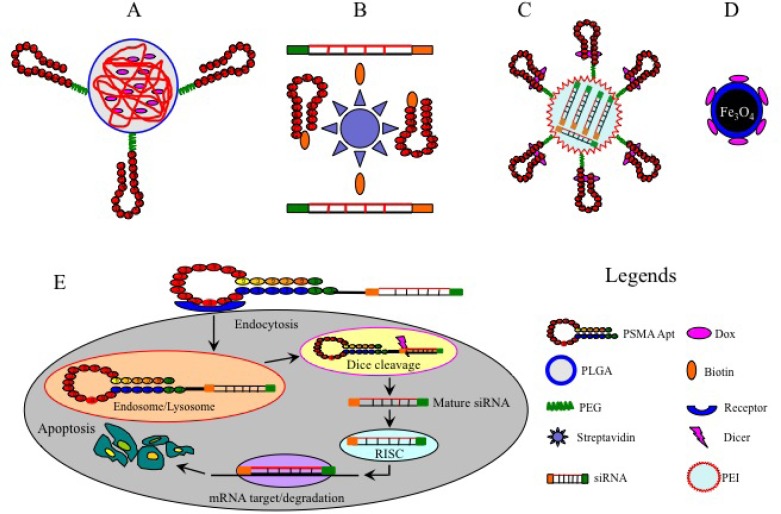
Examples of aptamer-mediated delivery of therapeutic agents **A.**, Dox-encapsulated PLGA-PEG-A10 bioconjugates; **B.**, A9-siRNA chimeras linked by a streptavidin bridge; **C.**, shRNA-coupled PEI-PEG-A10/DOX chimera; **D.**, Dox-coupled TCL-SPION-A10 complex; E, Specific delivery of siRNA-A10 chimera to target cancer cells. The A10 aptamer portion of the chimera binds to the cell surface receptor and is internalized *via* receptor-mediated endocytosis. After escaping from the endosome/lysosome, the chimera is separated by Dicer and the mature siRNA component against the BCL-2 gene is released and further incorporated into RISC, leading to the silencing of target mRNA and subsequent cancer cell apoptosis.

Efficient and selective delivery of therapeutic agents to tumor sites is always a challenge in cancer therapy. The off-target effects of the therapeutic reagents are responsible for commonly encountered adverse effects such as hair loss, nausea, myelosuppression and cardiotoxicity [8], and may lead to treatment failure. In order to overcome these off-target effects, cytotoxic agents can be conjugated to aptamers which can selectively deliver the loaded drugs to tumor cells, therefore avoiding the non-specific uptake of therapeutic agents by noncancerous cells (Table 3, Figure 2A).

**Table 3:** Aptamers (apt) as delivery tools for therapeutic agents

Cancer type	Apt (DNA/RNA)	Targets	Therapeutic agents	Linkage	Delivery vehicle	Trial state
PC	A10 (RNA)	PSMA	Dox	Intercalation	A10	*In vitro* [81]
	A10 (RNA)	PSMA	Dtxl/Cisplatin	Encapsulation	PLGA-b-PEG NPs	*In vitro/In vivo* [84-86]
	A10 (RNA)	PSMA	Cisplatin and Dtxl	Encapsulation	PLA/PLGA-PEG NPs	*In vitro* [87]
	A9 (RNA)	PSMA	Dox	Intercalation	PAD	*In vitro/In vivo* [88]
	A9 (RNA)	PSMA	Dox	Encapsulation	liposome	*In vitro/In vivo* [89]
	A10 (RNA)	PSMA	Dox	Intercalation	TCL-SPIONS	*In vitro/In vivo* [90,91]
	A10 (RNA)	PSMA	Dox	Intercalation	QD	*In vitro* [92]
	A10 (RNA)	PSMA	PLK1/BCL-2 siRNA	Covalent	A10	*In vitro/In vivo* [93]
	A10 (RNA)	PSMA	PLK1 siRNA	Covalent	A10	*In vitro/In vivo* [94]
	A9 (RNA)	PSMA	Lamin A/C siRNA	Covalent	A9	*In vitro* [95]
	T-A10 (RNA)	PSMA	Upf2/Smg1 siRNA	Covalent	Truncated A10	*In vitro/In vivo* [96]
	T-A10 (RNA)	PSMA	DNAPK shRNA	Covalent	Truncated A10	*In vivo* [97]
	A10 (RNA)	PSMA	Bcl-xL shRNA	Covalent	PEI-PEG	*In vivo* [98]
	A10-3 (RNA)	PSMA	miR-15a/miR-16-1	Covalent	ATE	*In vitro/In vivo* [99]
Leukemia	Sgc8 (DNA)	PTK7	Dox	Hydrazone	Sgc8	*In vitro* [101]
	Sgc8 (DNA)	PTK7	Dau	Intercalation	Sgc8	*In vitro* [102]
	Nucleolin apt (DNA)	Nucleolin	β-arrestin apt	Covalent	Nucleolin apt	*In vivo* [103]
*Lymphoma*	BAFF-R apt (RNA)	BAFF-R	STAT3 siRNA	Covalent	Apt	*In vitro* [104]
	TD05 (DNA)	mIgM	PEG	Covalent	TD05	*In vitro/in vivo* [106]
LC	MA3 (DNA)	MUC1	Dox	Intercalation	MUC1 apt	*In vitro* [107]
	MUC1-apt (DNA)	MUC1	Plasmid DNA	Covalent	PEI	*In vitro* [108]
	Nucleolin-apt (RNA)	Nucleolin	SLUG/NRP1 siRNA	Covalent	Nucleolin apt	*In vivo* [109]
	GL21-T (RNA apt)	Axl	Let-7g miRNA	Covalent	GL21-T	*In vitro/In vivo* [110]
BC	HER2-apt (RNA)	HER2	Bcl-2 siRNA	Covalent	HER2 Apt	*In vitro* [111]
	HB5 (DNA)	HER2	Dox	Intercalation	HB5	*In vitro* [112]
	AS1411 (DNA)	Nucleolin	Dox	Covalent	Liposomes	*In vitro/In vivo* [113,114]
	AS1411 (DNA)	Nucleolin	Vinorelbine	Intercalation	PLGA-PEG NPs	*In vitro/In vivo* [115]
	MUC1-apt (DNA)	MUC1	Dox	Intercalation	Apt-PEG	*In vitro* [116]
	MUC1-apt (DNA)	MUC1	PTX	Encapsulation	PLGA NPs	*In vitro* [117]
	TfR-apt (RNA)	TfR	miR-126	Covalent	TfR apt	*In vitro* [118]
OC	MUC1-apt (DNA)	MUC1	Dox	Encapsulation	QD	*In vitro/In vivo* [119]
	MUC1-apt (DNA)	MUC1	miR-29b	Covalent	MUC1 Apt	*In vitro/In vivo* [120]
	MUC1-apt (DNA)	MUC1	PTX and let7i	Covalent	MUC1 Apt	*In vitro* [121]
CRC	5TR1 (DNA)	MUC1	Epirubicin	Intercalation	TCL-SPION	*In vitro* [122]
	MUC1-apt (DNA)	MUC1	SN38	Encapsulation	Chitosan NPs	*In vitro* [123]
HC	TLS11-a (DNA)	LH86	Dox	Intercalation	TLS11a-GC	*In vitro/In vivo* [124]
PAC	SQ2 (RNA)	ALPPL2	5-Fluoro-2′-deoxyuridine	Covalent	SQ2	*In vitro* [125]
	TfR apt (RNA)	TfR	Dox/NF-*κ*B decoy	Intercalation	RNA apt	*In vitro* [126]

Abbreviations: BAFF: *B-cell–activating factor*; Dau: Daunorubicin; DNAPK: DNA-activated protein kinase; Dox: Doxorubicin; Dtxl: Docetaxel; MUC1: Mucin1; PAC; pancreatic cancer; PAD: polyamidoamine dendrimer; PEG: polyethylene glycol; PEI: polyethylenimine; PLA: poly lactic acid; PLGA: poly (lactic-co-glycolic acid); PLK-1: polo-like kinase 1; PTK7: protein tyrosine kinase 7; PTX: paclitaxel; QD: quantum dot; *SLUG*: snail family zinc finger; TCLSPION: thermally cross-linked superparamagnetic iron oxide nanoparticles; TR: transferrin receptor

Aptamer-conjugated Doxorubicin (Dox) is a classical example of using an aptamer as a drug delivery strategy. In the aptamer-Dox complex, Dox can preferably intercalate into the GC pairs of DNA double strands of aptamers to form a therapeutic complex that has been reported to significantly inhibit the proliferation of PSMA positive cells [84]. However, this approach is flawed by greatly reduced drug loading efficiency and rapid systemic clearance of the complex. Over recent years, aptamer-guided nanoparticles (NPs) have been shown to greatly enhance the uptake and retention of therapeutic drugs in cancers. The aptamer-NP structures have several superior properties over traditional delivery systems: (i) the surfaces of NPs can be precisely modified and programmed to specifically recognize and attach to targets [85]; (ii) NPs can be made sufficiently large and structurally flexible to accommodate different drugs, resulting in high drug loading and synergistic anti-cancer effects [86]; (iii) NPs can be easily coated with macromolecules such as PEG to extend their half-life in circulation and hence bioavailability [86]; and (iv) NPs have the potential to bypass conventional drug resistance mechanisms since they enter cells through endocytosis [85]. As such, aptamer-based NPs have been extensively explored in targeted drug delivery. In the following sections, we will elaborate on the applications of aptamers as carriers of single therapeutic agents or NPs-based drugs in a variety of cancers.

### Application of aptamer-based therapy in prostate cancer

PSMA is a type-II integral membrane glycoprotein that is abundantly expressed in most cases of prostate cancer, and therefore has become a promising therapeutic target for prostate cancer [84]. Aptamers A9 and A10 against extracellular domains of PSMA have been extensively studied. The most noteworthy examples are biocompatible and biodegradable poly (lactic-co-glycolic acid) (PLGA)-PEG NPs, which are surface-coated with A9 and A10 to enable the specific delivery of chemotherapeutic agents to prostate cancer cells (Figure 2A). These bioconjugates effectively target cancer cells and trigger sustained intracellular drug release, leading to enhanced anti-tumor effects and reduced toxicity to non-target cells *in vitro* and *in vivo* compared to free drugs and drug-NP conjugates lacking PSMA aptamers [87-89].

However, single chemotherapy drug regimens are often inadequate to eradicate tumor cells due to complicated chemo-resistance mechanisms. To overcome this problem, a dual-drug delivery NP system constructed by linking cisplatin-coupled poly lactic acid (PLA) polymer and Docetaxel (Dtxl)-encapsulated PLGA-PEG has been developed. This complex was further armed with A10, which allows specific and efficient co-delivery of cisplatin and Dtxl to prostate cancer cells, leading to superior synergistic anti-tumor efficacy over single drug-NP counterparts *in vitro* [90]. Furthermore, a different type of nanostructure was assembled by connecting a polyamidoamine dendrimer (PAD) to a single-stranded oligonucleotide which was then subsequently annealed to a Dox-intercalated A9 aptamer [91]. The resultant chimeras exerted a greater anti-tumor activity *in vitro* and *in vivo* compared to free Dox or A9-free dendrimer conjugates. A recently designed novel A9-conjugated liposome was used to encapsulate Dox and the resulting complex was found to induce significant cytotoxicity to prostate cancer cells *in vitro* and *in vivo* [92]. Moreover, certain NPs can have a dual function for cancer diagnosis and drug delivery. For example, Dox-coupled A10 aptamer can be conjugated with imaging agents such as thermally cross-linked superparamagnetic iron oxide nanoparticles (TCL-SPION) (Figure 2D) and quantum dot (QD) which can be tracked by computed tomography or magnetic resonance imaging (MRI), allowing real-time monitoring of drug transportation to tumor cells [93-95].

PSMA aptamers have also been utilized to deliver therapeutic nucleic acids such as small interfering RNAs (siRNAs), small hairpin RNAs (shRNAs) and microRNAs (miRNAs). In this regard, aptamer-mediated delivery of therapeutic siRNAs against anti-apoptosis genes polo-like kinase 1 (PLK-1) and B-cell lymphoma 2 (BCL-2) has been tested in prostate cancer cells with encouraging results [96] (Figure 2E). Modification of these therapeutic chimeras such as truncation at the aptamer portion of A10-PLK1 chimera and addition of PEG led to enhanced apoptosis *in vitro* and a greater inhibitory effect on tumor growth *in vivo* [97]. Other modifications to the aptamer-mediated delivery of therapeutic nucleic acids have been reported. For example, a streptavidin bridge was used as a chemical linker to conjugate biotinylated A9 aptamer with two biotinylated siRNAs against lamin A/C and GAPDH (Figure 2B). The disulfide linker between siRNA and biotin can be cleaved upon the internalization of aptamer-bound siRNAs, resulting in the silencing of target genes in target cells [98]. However, this chimera was found to be associated with *in vivo* immunogenicity that was provoked by streptavidin, and this drawback may hamper its clinical application. Additionally, truncated A10 (T-A10) was used to successfully deliver siRNAs against Upf2 and Smg1 genes, two critical components of the surveillance mechanism called non-sense mRNA decay, in prostate cancer cells. This approach led to a significant reduction of Upf2 and Smg1 expression and the up-regulation of tumor rejection antigens, thereby inhibiting tumor growth *in vitro* and *in vivo* [99].

Apart from siRNAs, PSMA aptamers have been used as carriers of shRNAs and miRNAs. A truncated A10 aptamer conjugated to shRNA against DNA-activated protein kinase (DNAPK) (a key factor responsible for DNA repair in tumor cells), and this chimera could greatly sensitize prostate cancer cells to ionizing radiation-induced tumoricidal effect *in vivo* [100]. In another study, BCL-xL shRNA was encapsulated inside a polyethylenimine (PEI)-PEG complex that was pre-conjugated by DOX-intercalated A10 (Figure 2C), yielding a multi-functional chimera (shRNA/PEI-PEG-A10/DOX) that could induce massive death of PSMA (+) cells *in vitro* [101]. Additionally, the delivery of two tumor suppressors (miR-15a and miR-16-1) to PSMA (+) cells *via* the atelocollagen-conjugated A10-3 aptamer displayed superior anticancer effects over miRNA/ATE alone [102]. These findings highlight the improved therapeutic potential of aptamer-siRNA/shRNA/miRNA chimeras for prostate cancer.

### Application of aptamer-based therapy in leukemia

Protein tyrosine kinase 7 (PTK7) is over-expressed in approximately 70% of T cell acute lymphocytic leukemia (T-ALL) [103]. Aptamer sgc8 that specifically targets PTK7 can selectively bind to T-ALL cells with high affinity even if the malignant cells are mixed with hematopoietic cells or clinical samples [103]. When covalently conjugated with Dox or Daunorubicin (Dau), sgc8 showed similar cytotoxic effects on T-ALL cells to free drugs but with minimal toxicity on non-target cells [104, 105].

Recently, a novel aptamer targeting the scaffolding protein β-arrestin that interacts with numerous critical oncogenic signaling pathways was identified. This aptamer was capable of inhibiting the formation of the complex composed of β-arrestin and extracellular signal-regulated kinases (ERK), resulting in blockage of the hedgehog/smoothened and wingless/Frizzled pathways simultaneously. Moreover, selective delivery of this therapeutic aptamer into chronic myelogenous leukemia cells led to a significant inhibition of tumor growth [106].

### Application of aptamer-based therapy in lymphoma

B-cell-activating factor (BAFF) is a member of the TNF family of cytokines and is often exclusively over-expressed in B-cell malignancies such as non-Hodgkin's lymphoma (>NHL). The binding of BAFF to its receptor (BAFF-R) is critical for B cell proliferation and survival, rendering BAFF-R an attractive therapeutic target for lymphoma [107]. A RNA aptamer specific to BAFF-R showed strong inhibitory effects on BAFF-mediated B cell proliferation. Moreover, this aptamer carrying siRNA of signal transducer and activator of transcription 3 (STAT3) significantly reduced STAT3 activity in NHL cells [107]. Given that down-regulation of STAT3 can inhibit the growth of lymphoma [108], an anti-BAFF aptamer may serve as a promising dual-function therapeutic reagent for NHL. Additionally, the multimeric version of TD05 aptamer was shown to specifically bind to B-cell receptor (BCR) on lymphoma cells, and when coupled with a PEG linker, the affinity was greatly enhanced [109] *in vitro* and *in vivo*. Thus, this system may constitute an ideal drug delivery vehicle for lymphoma.

### Application of aptamer-based therapy in lung cancer

Mucin1 (MUC1) is a large cell surface transmembrane glycoprotein that is vital for cell adhesion and metastasis. Aberrant expression and mutation of MUC1 is frequently associated with the invasion and metastasis of many cancers including those of the colon, breast, ovary, and prostate, making it a potential therapeutic target [110]. Anti-MUC1 aptamers were developed to selectively deliver Dox or therapeutic plasmid DNA to MUC1 (+) lung cancer cells [110, 111]. In a recent study, two siRNAs of snail family zinc finger (SLUG) and neuropilin-1 (NRP1) which are known to be important transcriptional factors in the activation of key signaling pathways were successfully carried into lung cancer cells by an anti-nucleolin aptamer [112]. These agents caused specific knockdown of SLUG and NRP1 genes resulting in the suppression of angiogenesis and growth of lung cancer [112].

In addition to siRNA, a RNA aptamer GL21.T was developed to carry tumor suppressor let-7g miRNA into cancer cells *via* binding to oncogenic receptor tyrosine kinase Axl. This agent could significantly inhibit the proliferation and migration of lung cancer cells, thereby leading to significant reduction of lung cancer growth *in vivo* [113].

### Application of aptamer-based therapy in breast cancer

HER-2 is one of the most common cell surface biomarkers for breast cancer. Its over-expression is associated with increased aggressiveness and poor prognosis [35]. Several HER-2 aptamers acting as the transporter of therapeutic agents including BCL-2 siRNA and Dox, exert selective cytotoxicity on HER-2 expressing cells [114, 115]. As a reliable carrier, aptamer AS1411 was functionalized with liposomes or PLGA-PEG NPs to enable specific delivery of anti-cancer drugs such as Dox or vinorelbine to breast cancer cells with significant anti-tumor efficacy [116-118]. Additionally, anti-MUC1 aptamers conjugated with PEG or PLGA NPs have also been utilized as the carriers of chemotherapeutic agents, rendering significant and specific toxicity to breast cancer [119, 120].

The miRNA-126 plays an essential part in vessel growth and is often down-regulated in breast cancer. On the other hand, restoration of miRNA-126 expression inhibited tumor growth and metastasis, suggesting that over-expression of miRNA-126 may be a promising therapeutic strategy for breast cancer [106]. As such, an aptamer against transferrin receptor (TfR), which is often over-expressed in various solid tumors, was used to deliver the precursor of miRNA-126 (pre-miR-126) to breast cancer cells, and this chimera was found to significantly reduce the proliferation and paracrine endothelial cell recruitment of breast cancer cells [121].

### Application of aptamer-based therapy in ovarian cancer

Mutations of MUC1 are present in more than 90% of late stage epithelial ovarian cancer patients and are involved in tumor metastasis [122]. Consequently, targeting aberrant MUC1 may be therapeutically relevant. A QD-MUC1-Dox conjugate has been developed that is composed of anti-MUC1 aptamer and QD coupled with Dox *via* a hydrazone bond. The resulting construct showed stronger and specific cytotoxicity to ovarian cancer cells compared to free Dox [122]. In another study, an anti-MUC1 aptamer was used to deliver tumor suppressor miR-29b to ovarian cancer cells, leading to decreased chemo-resistance and a significant inhibition of tumor growth possibly *via* attenuating phosphatase and tensin homolog (PTEN) and mitogen-activated protein kinases-4 (MAPK4) signaling [123]. Additionally, MUC1 aptamer was able to specifically deliver let-7i miRNA to ovarian cancer cells, rendering these cells more sensitive to paclitaxel [124].

### Application of aptamer-based therapy in colorectal cancer

MUC1 aptamer-based NPs have been utilized to deliver therapeutic agents to cells or tissues. In a recent study, the conventional chemotherapeutic agent Epirubicin (Epi) was intercalated into an anti-MUC1 aptamer (5TR1) that was pre-conjugated to the surface of TCL-SPION to form an Epi-Apt-SPION tertiary complex. The resulting conjugate showed cytotoxic effects similar to Epi alone towards colorectal cancer cells but with much less toxicity to non-target cells [125].

SN-38 is an active metabolite of the chemotherapeutic agent irinotecan and shows 100-1000 times more anti-tumor potency than its pro-drug for colorectal cancer [126]. However, its poor solubility and high toxicity has restricted clinical application. In order to overcome these limitations, a biocompatible polymeric carrier chitosan which can improve solubility and biocompatibility was utilized to covalently link with SN-38 and further conjugated with an anti-MUC-1 aptamer for specific targeting, leading to enhanced anti-proliferation and increased efficacy of SN-38 on colorectal cancer cells [126].

### Application of aptamer-based therapy in hepatocellular carcinoma

A DNA aptamer termed TLS11a-GC was designed to specifically target hepatocellular carcinoma (HCC) cells [127]. When conjugated with Dox, the aptamer complex was found to induce significant apoptosis in HCC cells both *in vitro* and *in vivo*, making it a potential candidate for targeted drug delivery [127]. In a very recent study, AS1411 was conjugated with Dox using formaldehyde as a crosslinking agent. This AS1411-Dox adduct was able to efficiently deliver Dox to HCC cells and inhibit HCC growth *in vivo* with a similar efficiency achieved by free Dox but with much reduced side effects [128].

### Application of aptamer-based therapy in pancreatic cancer

The abnormal expression of alkaline phosphatase placental-like 2 (ALPPL2) in pancreatic cancer cells makes ALPPL2 a putative biomarker for the development of targeted therapy for pancreatic cancer [129]. Recently, a RNA aptamer SQ2 targeting ALPPL2 has been used to specifically deliver 5-fluoro-2′-deoxyuridine to pancreatic cancer cells and successfully inhibit the proliferation of recipient cells *in vitro* [129].

Acquired chemoresistance has been the main hurdle of chemotherapy in cancers. The nuclear factor κB (NF-κB) is a ubiquitous transcription factor and is frequently involved in chemo-resistance [130]. In order to improve the efficiency of chemotherapeutic agents, a RNA aptamer against TfR was used to selectively deliver Dox and an inhibitor of NF-*κ*B (decoy oligonucleotides) to pancreatic cancer cells. This assembly resulted in the targeted release of two payloads in the endolysosomal environment, effectively inhibited NF-*κ*B activity and ultimately enhanced Dox-mediated apoptosis in pancreatic cancer [130].

## CONCLUSIONS, CHALLENGES, AND FUTURE PERSPECTIVES

Aptamers possess numerous advantages over conventional antibodies such as smaller size, higher stability, ease of modification, and lack of immunogenicity. These properties have rendered aptamers promising tools for targeted cancer therapy. The encouraging data for some aptamers currently under clinical trials (such as AS1411 and NOX-A12) have opened promising avenues for apatmer-based molecular cancer therapy. By physical modifications such as conjugation with various NPs, aptamers can be used as a specific anticancer drug delivery method with efficient tumor penetration and less toxic effects. Furthermore, aptamers can be used to selectively deliver multiple therapeutic RNA reagents to tumors, implying that the combination of aptamer-based chemotherapy and RNAi technology may synergistically enhance therapeutic efficacy.

Despite the huge potential of aptamer-based cancer therapy, aptamers have some disadvantages such as serum instability and rapid renal clearance which may limit their clinical applicability. Consequently, various biochemical modifications of aptamers have been developed. For example, replacing 2′-hydroxyl of the ribose sugars with 2′-fluoro, 2′-NH_2_ (amino) and 2′-O-methyl (O-Me) was found to enhance the nuclease resistance and PK profiles of aptamers *in vivo* (23, 37, 39). Moreover, numerous bulky chemicals such as PLGA, PEG, liposomes, streptavidin or cholesterol have been utilized to conjugate aptamers so that the systemic clearance of the aptamers is reduced and their *in vivo* half-life can be considerably prolonged (87, 91-94). With these modifications, we envisage that aptamer-based therapy will offer superior therapeutic benefits to cancer patients. Further studies to identify specific molecular targets and large scale clinical trials testing the therapeutic effects of aptamers are expected to accurately assess their clinical potential for cancer therapy.

## References

[1] Partha R, Kristi DV, Erin ES, Rebecca S (2013). Application of aptamers for targeted theraputics. Arch Immunol Ther Exp.

[2] Pei XY, Zhang J, Liu J (2014). Clinical applications of nucleic acid aptamers in cancer. Mol Clin Oncol.

[3] Oney S, Lam RT, Bompiani KM, Blake CM, Quick G, Heidel JD, Liu JY, Mack BC, Davis ME, Leong KW, Sullenger BA (2009). Development of universal antidotes to control aptamer activity. Nat Med.

[4] Zhu GZ, Mao Y, Michael J, Song E, Zhao Z, Tan W (2012). Nucleic acid aptamers: an emerging frontier in cancer therapy. Chem Commun (Camb).

[5] Joseph AP, Dalia LC, Zhi Z, Xu Y (2008). Applications of aptamers in cancer cell biology. Anal Chim Acta.

[6] Dhua PA, Kimarst S, Lee D (2011). Nucleic acid aptamers targeting cell-surface proteins. Methods.

[7] Muhammed A, Kwang G (2007). Drug-target network. Nat Biotechnol.

[8] Ng EW, Shima DT, Calias P, Adamis AP (2006). Pegaptanib, a targeted anti-VEGF aptamer for ocular vascular disease. Nat Rev Drug Discov.

[9] Alvarez RH, Kantarjian HM, Cortes JE (2006). Biology of platelet-derived growth factor and its involvement in disease. Mayo Clin Proc.

[10] Jo N, Mailhos C, Ju M, Cheung E, Bradley J, Nishijima K, Robinson GS, Adamis AP, Shima DT (2006). Inhibition of platelet-derived growth factor B signaling enhances the efficacy of anti-vascular enclothelial growth factor therapy in multiple models of ocular neovascularization. Am J Pathol.

[11] Akiyama H, Kachi S, Silva RL, Umeda N, Hackett SF, McCauley D, McCauley T, Zoltoski A, Epstein DM, Campochiaro PA (2006). Intraocular injection of an aptamer that binds PDGFB: a potential treatment for proliferative retinopathies. J Cell Physiol.

[12] Sennino B1, Falcón BL, McCauley D, Le T, McCauley T, Kurz JC, Haskell A, Epstein DM, McDonald DM (2007). Sequential loss of tumour vessel pericytes and endothelial cells after inhibition of platelet-derived growth factor B by selective aptamer AX102. Cancer Res.

[13] Kaur H, Li JJ, Bay BH (2013). Investigating the anti-proliferative activity of high affinity DNA aptamer on cancer cells. PLoS One.

[14] Stacey LE, Vasanthanathan P, Jagat RK, Kislay R, Kristine MH, Neerati P (2015). Targeting VEGF with LNA-stabilized G-rich oligonucleotide for efficient breast cancer inhibition. Chem Commun.

[15] Kwak H, Hwang I, Kim JH, Kim MY, Yang JS, Jeong S (2009). Modulation of transcription by the peroxisome proliferator-activated receptor δ-binding RNA aptamer in colon cancer cells. Mol Cancer Ther.

[16] Cahristdropher RI, Lloyd RK (2006). Discovery and development of anticancer aptamers. Mol Cancer Ther.

[17] Bates PJ, Laber DA, Miller DM, Thomas SD, Trent JO (2009). Discovery and development of the G-rich oligonucleotide AS1411 as a novel treatment for cancer. Exp Mol Pathol.

[18] Mongelard F, Bouvetety P (2010). AS 1411, a guanosine rich oligo¬nucleotide aptamer targeting nucleolin for the potential treatment of cancer, including acute myeloid leukemia. Curr Opin Mol Ther.

[19] Rosenberg JE, Bambury RM, Van Allen EM, Drabkin HA, Lara PN, Harzstark AL, Wagle N, Figlin RA, Smith GW, Garraway LA, Choueiri T, Erlandsson F, Laber DA (2014). A phase II trial of AS1411 (a novel nucleolin-targeted DNA aptamer) in metastatic renal cell carcinoma. Invest New Drugs.

[20] Marasca R (2014). NOX-A12: Mobilizing CLL away from home. Blood.

[21] Vater A, Sahlmann J, Kroger N, Zollner S, Lioznov M, Maasch C, Buchner K, Vossmeyer D, Schwoebel F, Purschke WG, Vonhoff S, Kruschinski A, Hubel K, Humphrey M, Klussmann S, Fliegert F (2013). Hematopoietic stem and progenitor cell mobilization in mice and humans by a first-in-class mirror-image oligonucleotide inhibitor of CXCL12. Clin Pharmacol Ther.

[22] Liu SC, Alomran R, Chernikova SB, Lartey F, Stafford J, Jang T, Merchant M, Zboralski D, Zollner S, Kruschinski A, Klussmann S, Recht L, Brown JM (2014). Blockade of SDF-1 after irradiation inhibits tumor recurrences of autochthonous brain tumors in rats. Neuro Oncol.

[23] Hinterseer E (2013). The spiegelmer NOX-A12 abrogates homing of human CLL cells to bone marrow and mobilizes murine CLL cells in the Eu-TCL1 transgenic mouse model of CLL. American Society of Hematology.

[24] Hoellenriegel J, Zboralski D, Maasch C, Rosin NY, Wierda WG, Keating MJ (2014). The spiegelmer NOX-A12, a novel CXCL12 inhibitor, interferes with chronic lymphocytic leukemia cell motility and causes chemosensitization. Blood.

[25] Zhang H, Berezov A, Wang Q, Zhang G, Drebin J, Murali R, Greene MI (2007). ErbB receptors: from oncogenes to targeted cancer therapies. J Clin Invest.

[26] Chen CH, Chernis GA, Hoang VQ, Landgraf R (2003). Inhibition of heregulin signaling by an aptamer that preferentially binds to the oligomeric form of human epidermal growth factor receptor-3. Proc Natl Acad Sci USA.

[27] Li N, Nguyen HH, Byrom M, Ellington AD (2011). Inhibition of cell proliferation by an anti-EGFR aptamer. PLoS One.

[28] Esposito CL, Passaro D, Longobardo I, Condorelli G, Marotta P, Affuso A, Franciscis V, Cerchia L (2011). A neutralizing RNA aptamer against EGFR causes selective apoptotic cell death. PLoS One.

[29] Wan Y, Tamuly D, Allen PB, Kim YT, Bachoo R, Ellington AD, Iqbal SM (2013). Proliferation and migration of tumor cells in tapered channels. Biomed Micro.

[30] Buerger C, Nagel WK, Kunz C, Wittig I, Hoppe-Seyler F, Groner B (2003). Sequence-specific peptide aptamers, interacting with the intracellular domain of the epidermal growth factor receptor, interfere with Stat3 activation and inhibit the growth of tumor cells. J Biol Chem.

[31] Wang DL, Song YL, Zhu Z, Li XL, Zou Y, Yang HT, Wang JJ, Yao PS, Pan RJ, Yang CJ, Kang DZ (2014). Selection of DNA aptamers against epidermal growth factor receptor with high affinity and specificity. Biochem Biophys Res Commun.

[32] Tan Y, Liang H, Wu X, Gao Y, Zhang X (2013). Cell-ELA-based determination of binding affinity of DNA aptamer against U87-EGFRvIII cell. Sheng Wu Gong Cheng Xue Bao.

[33] Wu XD, Liang HY, Tan Y, Yuan C, Li S, Li X, Li G, Shi Y, Zhang Y (2014). Cell-SELEX aptamer for highly specific radio nuclide molecular imaging of glioblastoma *in vivo*. PLoS One.

[34] Simona C, Elvira C, David C, Andrea C, Angela A, Monica F, Mario C, Laura C (2015). Aptamer targeting EGFRvIII mutant hampers its constitutive autophosphorylation and affects migration, invasion and proliferation of glioblastoma cells. Oncotarget.

[35] Kim MY, Jeong SJ (2011). *In vitro* selection of RNA aptamer and specific targeting of ErbB2 in breast cancer cells. Nucleic Acid Ther.

[36] Georg M, Ruth M, Maicol M, Schechter B, Sela M, Yarden Y (2013). Aptamer to ErbB-2/HER2 enhances degradation of the target and inhibits tumourigenic growth. Proc Natl Acad Sci USA.

[37] Peng L, Valerie MW, Zena W (2012). The extracellular matrix: A dynamic niche in cancer progression. J Cell Biol.

[38] Liu GX, Xi HQ, Sun XY, Wei B (2015). Role of periostin and its antagonist PNDA-3 in gastric cancer metastasis. World J Gastroenterol.

[39] Lee YJ, Kim IS, Park SA, Kim Y, Lee JE, Noh DY, Kim KT, Ryu SH, Suh PG (2013). Periostin-binding DNA Aptamer Inhibits Breast Cancer Growth and Metastasis. Mole Ther.

[40] Brian JH, Andrew WS, Chang YF, Cynthia KL, James H, Sandra B (2006). Tumor targeting by an aptamer. J Nucl Med.

[41] Daniels DA, Chen H, Hicke BJ, Swiderek KM, Gold L (2003). A tenascin-C aptamer identified by tumour cell SELEX: systematic evolution of ligands by exponential enrichment. Proc Natl Acad Sci USA.

[42] Hicke BJ, Marion C, Chang YF, Gould T, Lynott CK, Parma D, Schmidt PG, Warren S (2001). Tenascin-C aptamers are generated using tumour cells and purified protein. J Biol Chem.

[43] Cesar SF, Edna TK (2015). Insights into regulation of the miR-17-92 cluster of miRNAs in cancer. Front Med.

[44] Christina EL, Gracjan M, Christine SH, Andrea R, Javier FC, Michael F (2010). An aptamer targeting the apical-loop domain modulates pri-miRNA processing. Angew Chem Int Ed.

[45] Nithya S, Rupinder KK, Subramanian K (2015). Blocking the maturation of oncomiRNAs using pri-miRNA-17-92 aptamer in retinoblastoma. Nuleic Acid Ther.

[46] Zamay TN, Kolovskaya OS, Glazyrin YE, Zamay GS, Kuznetsova SA, Spivak EA, Wehbe M, Savitskaya AG, Zubkova OA, Kadkina A, Wang X, Muharemagic D, Dubynina A (2014). DNA-aptamer targeting vimentin for tumor therapy *in vivo*. Nucleic Acid Ther.

[47] Lee YJ, Seung RH, Kim NY, Lee SH, Jeong JS, Lee SW (2012). An RNA aptamer that binds carcinoembryonic antigen inhibits hepatic metastasis of colon cancer cells in mice. Gastroenterology.

[48] Lee YJ, Lee SW (2012). Regression of hepatocarcinoma cells using RNA aptamer specific to alpha-fetoprotein. Bio Biophy Res Com.

[49] Mern DS, Hasskarl J, Burwinkel B (2010). Inhibition of Id proteins by a peptide aptamer induces cell-cycle arrest and apoptosis in ovarian cancer cells. Br J Cancer.

[50] Mern DS, Hoppe-Seyler K, Hoppe-Seyler F (2010). Targeting Id1 and Id3 by a specific peptide aptamer induces E-box promoter activity, cell cycle arrest, and apoptosis in breast cancer cells. Breast Cancer Res Treat.

[51] Ojima A, Matsui T, Maeda S, Takeuchi M, Inoue H, Higashimoto Y, Yamagishi S (2014). DNA aptamer raised against advanced glycation end products inhibits melanoma growth in nude mice. Lab Invest.

[52] Mi J, Zhang X, Rabbani ZN, Liu Y, Reddy SK, Su Z, Salahuddin FK, Viles K, Giangrande PH, Dewhirst MW, Sullenger BA, Kontos CD, Clary BM (2008). RNA aptamer-targeted inhibition of NF-κB suppresses non-small cell lung cancer resistance to Doxorubicin. Mol Ther.

[53] Cerchia L, Esposito CL, Camorani S, Rienzo A, Stasio L, Insabato L (2012). Targeting axl with an high-affinity inhibitory aptamer. Mol Ther.

[54] Mi ZY, Guo HT, Russel MB, Liu Y, Sullenger BA, Kuo PC (2009). RNA aptamer blockade of osteopontin inhibits growth and metastasis of MDA-MB231 breast cancer Cells. Mol Ther.

[55] Talbot LJ, Mi ZY, Bhattacharya SD, Kim V, Guo H (2011). Pharmacokinetic characterization of an RNA aptamer against osteopontin (OPN) and demonstration of *in vivo* efficacy in reversing growth of human breast cancer cells. Surgery.

[56] Zhang K, Sefah K, Tang L, Zhao Z, Zhu G, Ye M, Sun W, Goodison S, Tan W (2012). A novel aptamer developed for breast cancer cell internalization. Chem Med Chem.

[57] Zhang XJ, Zhang J (2014). A cell-based single-stranded DNA aptamer specifically targets gastric cancer. Int J Bio Cell Bio.

[58] Cao HY, Yuan AH, Chen W, Shi XS, Miao Y (2014). A DNA aptamer with high affinity and specificity for molecular recognition and targeting therapy of gastric cancer. BMC Cancer.

[59] Faryammanesh R, Lange T, Magbanua E, Haas S, Meyer C, Wicklein D, Schumacher U, Hahn U (2014). SDA, a DNA aptamer inhibiting E- and P-selectin mediated adhesion of cancer and leukemia cells, the first and pivotal step in transendothelial migration during metastasis formation. PLoS One.

[60] Dimitri VS, Diane T, Carole C (2014). Identification of cell membrane protein stress-induced phosphoprotein 1 as a potential ovarian cancer biomarker using aptamers selected by cell systematic evolution of ligands by exponential enrichment. Anal Chem.

[61] Li SH, Wang W, Ding HM, Xu H, Zhao Q, Li J (2012). Aptamer BC15 against heterogeneous nuclear ribonucleoprotein A1 has potential value in diagnosis and therapy of hepatocarcinoma. Nucleic Acid Ther.

[62] Kim YH, Sung HJ, Kim S, Kim EO, Lee JW, Moon JY, Choi K, Jung JE, Lee Y, Koh SS, Rhee SG, Heo K, Kim IH (2011). A RNA aptamer that specifically binds pancreatic adenocarcinoma up-regulated factor inhibits migration and growth of pancreatic cancer cells. Cancer Lett.

[63] Dassie JP, Hernandez LI, Thomas GS, Long ME, Rockey WM, Howell CA, Chen Y, Hernandez FJ, Liu XY, Wilson ME, Allen LA, Vaena DA, Meyerholz DK, Giangrande PH (2014). Targeted inhibition of prostate cancer metastasis with an RNA aptamer to prostate-specific membrane antigen. Mol Ther.

[64] Svobodova M, Bunka DH, Nadal P, Stockley PG, OSullivan CK (2013). Selection of 2′F-modified RNA aptamers against prostate-specific antigen and their evaluation for diagnostic and therapeutic applications. Anal Bioanal Chem.

[65] Suzanne CS, Hannaleena J, Dilson S, Celia MC, Edward AM (2014). Anti-heparanase aptamers as potential diagnostic and therapeutic agents for oral cancer. PLoS One.

[66] Borghouts B, Delis N, Brill B, Weiss A, Mack L, Lucks P, Groner B (2012). A membrane penetrating peptide aptamer inhibits STAT3 function and suppresses the growth of STAT3 addicted tumor cells. Mol Ther.

[67] Roald N (2014). Aptamers in immunological research. Immunol Lett.

[68] Eli G, Fernando P (2013). Use of oligonucleotide aptamer ligands to modulate the function of immune receptors. Clin Cancer Res.

[69] Mcnamjhfara JO, Kolonias D, Pastor F, Mittler RS, Chen L, Giangrande PH, Sullenger B, Gilboa E (2008). Multivalent 4-1BB binding aptamers costimulate CD8+ T cells and inhibit tumour growth in mice. J Clin Invest.

[70] Dollins CM, Nair S, Boczkowski D, Lee J, Layzer JM, Gilboa E, Sullenger BA (2008). Assembling OX40 aptamers on a molecular scaffold to create a receptor-activating aptamer. Chem Biol.

[71] Elizabeth DP, Bruce AS, Smita KN (2013). Identification and characterization of an agonistic aptamer against the T cell costimulatory receptor OX40. Nucleic Acid Ther.

[72] Fernando P, Mario MS, Helena V, Kolonias D, Inoges S, Cerio AL (2013). CD28 aptamers as powerful immune response modulators. Mol Ther Nucl Acids.

[73] Santuli MS, Nair SK, Rusconi C, Sullenger B, Gilboa E (2003). Multivalent RNA aptamers that inhibit CTLA-4 and enhance tumour immunity. Cancer Res.

[74] Pastorv F, Kolonias D, McNamara JO (2011). Targeting 4-1BB costimulation to disseminated tumor lesions with bi-specific oligonucleotide aptamers. Mol Ther.

[75] Eder JP, Vande WF, Boerner SA, Lorusso PM (2009). Novel therapeutic inhibitors of the c-Met signaling pathway in cancer. Clinical Can Res.

[76] Schrand B, Berezhnoy A, Brenneman R, Williams A, Levay A, Kong LY, Rao G, Zhou S, Heimberger AB, Gilboa E (2014). Targeting 4-1BB costimulation to the tumour stroma with bispecific aptamer conjugates enhances the therapeutic index of tumour immunotherapy. Cancer Immunol Res.

[77] Massari F, Santoni M, Ciccarese C, Santini D, Alfieri S, Martignoni G, Brunelli M, Piva F, Berardi R, Montironi R, Porta C, Cascinu S (2015). PD-1 blockade therapy in renal cell carcinoma: Current studies and future promises. Cancer Treat Rev.

[78] Borghaei H, Horn L, Spigel D, Steins M, Ready NE, Chow LQ, Vokes EE, Felip E, Holgado E, Barlesi F, Kohlhaufl M, Arrieta O (2015). Nivolumab *versus* docetaxel in advanced nonsquamous non-small-cell lung cancer. N Engl J Med.

[79] Aaron P, Aws AW, Nicholas WF, Eric HB, Marzena C, Jean G (2015). Targeting the PD-1/PD-L1 immune evasion axis with DNA aptamers as a novel therapeutic strategy for the treatment of disseminated cancers. Mol Ther Nucl Acids.

[80] Berezhnoy A, Stewart CA, Mcnamara JO, Thiel W, Giangrande P, Trinchieri G, Gilboa E (2012). Isolation and optimization of murine IL-10 receptor blocking oligonucleotide aptamer using high-throughput sequencing. Mol Ther.

[81] Roth F, Dea FC, Vella JL, Zoso A, Inverardi L, Serafini P (2012). Aptamer-mediated blockade of IL4Rα triggers apoptosis of MDSCs and limits tumour progression. Cancer Res.

[82] Gupta S, Hirota M, Waugh SM, Murakami I, Suzuki T, Muraguchi M, Shibamori M, Ishikawa Y, Jarvis TC, Carter JD, Zhang C, Gawande B, Vrkljan M, Janjic N, Schneider DJ (2014). Chemically modified DNA aptamers bind interleukin-6 with high affinity and inhibit signaling by blocking its interaction with interleukin-6 receptor. J Biol Chem.

[83] Erik WO, Nick J, Yuen LS, Sachdev SS, Jean G (2013). A Short DNA aptamer that recognizes TNF-α and blocks its activity *in vitro*. ACS Chem Biol.

[84] Bagalkot V, Omid CF, Robert L, Jon S (2006). An aptamer-doxorubicin physical conjugate as a novel targeted drug-delivery platform. Angew Chem Int Ed.

[85] Yang L, Zhang X, Ye M, Jiang J, Yang R (2011). Aptamer-conjugated nanomaterials and their applications. Adv Drug Deliv Rev.

[86] Wu C, Han D, Chen T (2013). Building a multifunctional aptamer-based DNA nanoassembly for targeted cancer therapy. J Am Chem Soc.

[87] Farokhzad OC, Cheng J, Teply BA, Sherifi I, Jon S, Kantoff PW, Richie JP, Langer R (2006). Targeted nanoparticle-aptamer bioconjugates for cancer chemotherapy *in vivo*. Proc Natl Acad Sci USA.

[88] Dhara S, Gub FX, Langer R, Farokhzad OC, Lippard SJ (2008). Targeted delivery of cisplatin to prostate cancer cells by aptamer functionalized Pt (IV) prodrug-PLGA-PEG nanoparticles. Proc Natl Acad Sci USA.

[89] Dhar S, Kolishetti N, Lippard SJ, Farokhzad OC (2011). Targeted delivery of a cisplatin prodrug for safer and more effective prostate cancer therapy *in vivo*. Proc Natl Acad Sci USA.

[90] Kolishetti N, Dhar S, Valencia PM, Lin LQ, Karnik R, Lippard SJ, Langer R, Farokhzad OC (2010). Engineering of self-assembled nanoparticle platform for precisely controlled combination drug therapy. Proc Natl Acad Sci USA.

[91] Lee IH, An S (2011). Targeted chemo-immunotherapy using drug-loaded aptamer-dendrimer bioconjugates. J Control Release.

[92] Baek SE, Lee KH, Park YS, Oh DK, Oh S, Kim KS, Kim DE (2014). RNA aptamer-conjugated liposome as an efficient anticancer drug delivery vehicle targeting cancer cells *in vivo*. J Controll Release.

[93] Andrew Z, Wang VB (2008). Superparamagnetic iron oxide nanoparticle-aptamer bioconjugates for combined prostate cancer imaging and therapy. Chem Med Chem.

[94] Yu MK, Kim D, Lee IH, So JS, Jeong YY, Jon S (2011). Image-guided prostate cancer therapy using aptamer-functionalized thermally cross-linked superparamagnetic iron oxide nanoparticles. Small.

[95] Bagalkot V, Zhang L, Levy-Nissenbaum E, Jon S, Kantoff PW, Langer R, Farokhzad OC (2007). Quantum dot-aptamer conjugates for synchronous cancer imaging, therapy, and sensing of drug delivery based on bi-fluorescence resonance energy transfer. Nano Lett.

[96] Mcnamara JO, Andrechek ER, Wang Y, Viles KD, Rempel RE, Gilboa E, Sullenger BA, Giangrande PH (2006). Cell type specific delivery of siRNAs with aptamer-siRNA chimeras. Nat Biotechnol.

[97] Dassie JP, Liu XY, Thomas GS (2009). Systemic administration of optimized aptamer-siRNA chimeras promotes regression of PSMA-expressing tumours. Nat Biotechnol.

[98] Chu TC, Twu KY, Ellington AD, Levy M (2006). Aptamer mediated siRNA delivery. Nucleic Acids Res.

[99] Pastorssaerr F, Kolonias D, Giangrande PH, Gilboa E (2010). Induction of tumour immunity by targeted inhibition of nonsense-mediated mRNA decay. Nature.

[100] Nidffe XH, Zhang YG, Judit R, Chowdhury WH, Castanares M, Zhang Z (2011). Prostate-targeted radiosensitization *via* aptamer-shRNA chimeras in human tumour xenografts. J Clin Invest.

[101] Kim E, Jung Y, Choi H, Yang J, Suh JS, Huh YM, Kim K, Haam S (2010). Prostate cancer cell death produced by the co-delivery of Bcl-xL shRNA and doxorubicin using an aptamer-conjugated polyplex. Biomaterials.

[102] Zhao H, Wei F, Jian H, Wu X, Zeng GQ, Zhang LJ, Nie SF, Wang XD (2014). Efficient delivery of micro-RNA to bone-metastatic prostate tumours by using aptamer-conjugated atelocollagen *in vitro* and *in vivo*. Drug Deliv.

[103] Shangguan D, Cao ZC, Li Y, Tan W (2007). Aptamers evolved from cultured cancer cells reveal molecular differences of cancer cells in patient samples. Clin Chem.

[104] Huang YF, Shangguan D, Liu HP (2009). Molecular assembly of an aptamer-drug conjugate for targeted drug delivery to tumour Cells. Chembiochem.

[105] Taghdisi SM, Abnous K, Fatemeh M, Behravan J (2010). Targeted delivery of daunorubicin to T-cell acute lymphoblastic leukemia by aptamer. J Drug Targeting.

[106] Kotula JW, Sun J, Li M, Pratico ED, Fereshteh MP, Ahrens DP, Sullenger BA, Kovacs JJ (2014). Targeted disruption of β-Arrestin 2-mediated signaling pathways by aptamer chimeras leads to inhibition of leukemic cell growth. PLoS One.

[107] Zhou J, Tiemann K, Chomchan P, Alluin J, Swiderski P, Burnett J, Zhang X, Forman S, Chen R, Rossi J (2013). Dual functional BAFF receptor aptamers inhibit ligand-induced proliferation and deliver siRNAs to NHL cells. Nucleic Acids Res.

[108] Lai R, Rassidakis GZ, Medeiros LJ, Leventaki V, Keating M, McDonnell TJ (2003). Expression of STAT3 and its phosphorylated forms in mantle cell lymphoma cell lines and tumors. J Pathol.

[109] Mallikaratch PR, Ruggiero A, Gardner JR, Kuryavyi V, Maguire WF, Heaney ML, McDevitt MR, Patel DJ, Scheinberg DA (2011). A multivalent DNA aptamer specific for the B-cell receptor on human lymphoma and leukemia. Nucleic Acids Res.

[110] Hu Y, Duan JH, Zhan O, Wang F, Lu X, Yang XD (2012). Novel MUC1 aptamer selectively delivers cytotoxic agent to cancer cells *in vitro*. PLoS ONE.

[111] Kurosaki T, Higuchi NY, Kawakami SG, Higuchi Y, Nakamura T, Kitahara T (2012). Self-assemble gene delivery system for molecular targeting using nucleic acid aptamer. Gene.

[112] Lai WY, Wang WY, Chang YC, Chang CJ, Yang PC, Peck K (2014). Synergistic inhibition of lung cancer cell invasion, tumour growth and angiogenesis using aptamer-siRNA chimeras. Biomaterials.

[113] Esposito CL, Cerchia L, Catuogno S, De Vita G, Dassie JP, Santamaria G, Swiderski P, Condorelli G, Giangrande PH (2014). Multifunctional aptamer-miRNA conjugates for targeted cancer therapy. Mol Ther.

[114] Thiel KW, Hernandez LI, Dassi J (2012). Delivery of chemo-sensitizing siRNAs to HER2+-breast cancer cells using RNA aptamers. Nucleic Acids Res.

[115] Liu Z, Duan JH, Song YM, Ma J, Wang FD, Lu X, Yang XD (2012). Novel HER2 aptamer selectively delivers cytotoxic drug to HER2-positive breast cancer cells *in vitro*. J Transl Med.

[116] Xing H, Tang L, Yang X, Hwang K, Wang W, Yin Q, Wong NY, Dobrucki LW, Yasui N, Katzenellenbogen JA, Helferich WG, Cheng J, Lu Y (2013). Selective delivery of an anticancer drug with aptamer-functionalized liposomes to breast cancer cells *in vitro* and *in vivo*. J Mater Chem B Mater Biol Med.

[117] Liao ZX, Chuang EY, Lin CC, Ho YC, Lin KJ, Cheng PY, Chen KJ, Wei HJ, Sung HW (2015). An AS1411 aptamer-conjugated liposomal system containing a bubble-generating agent for tumor-specific chemotherapy that overcomes multidrug resistance. J Control Release.

[118] Zhou W, Zhou Y, Wu J, Liu Z, Zhao H, Liu J, Ding J (2014). Aptamer-nanoparticle bioconjugates enhance intracellular delivery of vinorelbine to breast cancer cells. J Drug Target.

[119] Tan L, Neoh KG, Kang ET, Choe WS, Su X (2011). PEGylated anti-MUC1 aptamer-doxorubicin complex for targeted drug delivery to MCF7 breast cancer cells. Macromol Biosci.

[120] Yu C, Hu Y (2011). Novel aptamer-nanoparticle bioconjugates enhances delivery of anticancer drug to MUC1-positive cancer cells *in vitro*. PLoS One.

[121] Rohde JH, Weigand JE, Beatrix S, Stefanie D (2015). A universal aptamer chimera for the delivery of functional microRNA-126. Nucleic Acid Ther.

[122] Savla R, Taratula O, Garbuzenko O, Minko T (2011). Tumour targeted quantum dot-mucin 1 aptamer-doxorubicin conjugate for imaging and treatment of cancer. J Control Release.

[123] Dai F, Zhang YV, Zhu XG (2013). The anti-chemoresistant effect and mechanism of MUC1 aptamer-miR-29b chimera in ovarian cancer. Gynec Oncol.

[124] Liu N, Zhou C, Zhao J, Chen Y (2012). Reversal of paclitaxel resistance in epithelial ovarian carcinoma cells by a MUC1 aptamer-let-7i chimera. Cancer Invest.

[125] Jalalian SH, Taghdisi SM, Shahidi Hamedani N, Kalat SA, Lavaee P, Zandkarimi M, Ghows N, Jaafari MR, Naghibi S, Danesh NM, Ramezani M, Abnous K (2013). Epirubicin loaded super paramagnetic iron oxide nanoparticle-aptamer bioconjugate for combined colon cancer therapy and imaging *in vivo*. Euro J Pharm Sci.

[126] Sayari E, Dinarvand M (2014). MUC1 aptamer conjugated to chitosan nanoparticles, an efficient targeted carrier designed for anticancer SN38 delivery. Int J Pharm.

[127] Meng L, Yang L, Zhao X, Zhang L, Zhu H, Liu C, Tan W (2012). Targeted delivery of chemotherapy agents using a liver cancer-specific aptamer. PLoS One.

[128] Thu LT, Zhu GZ, Xiao XL, William P, Kwame S, Qun W, Tan WH, Chen L (2015). A synthetic aptamer-drug adduct for targeted liver cancer therapy. PLoS One.

[129] Pooja D, Sajeesh S, Soyoun K, Lee DK (2015). ALPPL2 Aptamer-mediated targeted delivery of 5-Fluoro-2-Deoxyuridine to pancreatic cancer. Nucleic Acid Ther.

[130] Porciani D, Tedeschi L, Marchetti L, Citti L, Piazza V, Beltram F, Signore G (2015). Aptamer-mediated codelivery of doxorubicin and NF-κB decoy enhances chemosensitivity of pancreatic tumor cells. Mol Ther-Nucl Acids.

